# Too Imperfect to Fall Asleep: Perfectionism, Pre-sleep Counterfactual Processing, and Insomnia

**DOI:** 10.3389/fpsyg.2018.01288

**Published:** 2018-08-07

**Authors:** Ralph E. Schmidt, Delphine S. Courvoisier, Stéphane Cullati, Rainer Kraehenmann, Martial Van der Linden

**Affiliations:** ^1^Cognitive Psychopathology and Neuropsychology Unit, Department of Psychology, University of Geneva, Geneva, Switzerland; ^2^Department of Psychiatry, Psychotherapy, and Psychosomatics, Psychiatric Hospital, University of Zurich, Zurich, Switzerland; ^3^Department of General Internal Medicine, Rehabilitation and Geriatrics, University of Geneva, Geneva, Switzerland; ^4^Swiss National Centre of Competence in Research “LIVES – Overcoming Vulnerability: Life Course Perspectives”, Institute of Demography and Socioeconomics, University of Geneva, Geneva, Switzerland

**Keywords:** affect, counterfactual thinking, counterfactual emotion, insomnia, perfectionism, personality, sleep disorders

## Abstract

Previous research suggests that certain dimensions of perfectionism are associated with insomnia. However, the exact processes whereby perfectionism may influence sleep have as yet remained unexplored. The present study tested the hypothesis that perfectionistic individuals are particularly prone to engage in counterfactual thinking and to experience counterfactual emotions (regret, shame, and guilt) at bedtime, which have been shown to impair sleep. One hundred eighty university students completed questionnaires on perfectionism, counterfactual processing, and insomnia severity. Analyses revealed that three dimensions of perfectionism were significantly related to insomnia severity: Concern over mistakes and doubts about action showed positive correlations, whereas organization showed a negative correlation. Moreover, the frequency of counterfactual thoughts and emotions at bedtime largely mediated the effects of these dimensions of perfectionism on insomnia severity. These findings highlight how personality-related patterns of behavior may translate into affective arousal at bedtime, thereby increasing the risk of insomnia.

## Introduction

Insomnia symptoms are widely spread in the general population. Indeed, 59% of adults in the United States experience at least one symptom of insomnia every night or almost every night according to a recent poll of the National Sleep Foundation (National Sleep Foundation, 2015). Empirical evidence suggests that insufficient sleep is more prevalent among women (Zhang and Wing, 2006) and may entail a wide range of consequences, including impaired concentration and memory, increased risk of accidents, heightened risks of anxiety, depression and other medical problems such as cardiovascular disease, more frequent use of healthcare institutions, augmented absenteeism at the workplace and increased mortality (Mai and Buysse, 2008; Li et al., 2016; Bos and Macedo, 2018).

Current etiological models of insomnia assign a central role to cognitive, emotional, and physiological hyperarousal (Espie, 2002, 2007; Harvey, 2002; Riemann et al., 2010). Three categories of factors that contribute to sleep-interfering arousal may be distinguished (Spielman and Glovinsky, 1991): predisposing factors (e.g., personality traits), precipitating factors (e.g., stressful life events), and perpetuating factors (e.g., maladaptive coping strategies). Regarding the first category, a consistent body of research suggests that several personality traits of the internalizing spectrum may predispose for and perpetuate insomnia, in particular neuroticism and anxiety (Vincent et al., 2009; van de Laar et al., 2010). More recently, it has been shown that certain personality traits of the externalizing spectrum may also represent risk factors for insomnia, for instance, impulsive urgency (e.g., Schmidt et al., 2010a).

A central personality construct in clinical research is perfectionism. Indeed, a coherent body of evidence suggests that perfectionism may function as a predisposing and perpetuating factor for a wide range of psychopathological states, including eating disorders, anxiety, and depression (Egan et al., 2011; Smith et al., 2018). Several studies have also explored the relations between perfectionism and insomnia (for an overview, see **Table 3**). Initial evidence came from an investigation by Lundh et al. (1994), who administered a short version of the Frost Multidimensional Perfectionism Scale (FMPS) (Frost et al., 1990) and questions on sleep to 383 individuals from the general population and to 70 patients with persistent insomnia. Frost et al. (1990) proposed that perfectionistic people may be characterized by the following six dimensions: personal standards (tendency to set high standards), concern over mistakes (tendency to react negatively to mistakes and to perceive mistakes as failures), doubts about action (tendency to doubt the quality of one’s own performance), parental expectations (tendency to perceive parents as setting high standards), parental criticism (tendency to perceive parents as being very critical), and organization (preference for precision, order, organization). Using these distinctions, Lundh et al. (1994) found that patients with insomnia obtained comparatively higher scores on the dimensions concern over mistakes, doubts about action, and personal standards. Moreover, these three dimensions of perfectionism were also positively correlated with sleep problems in the sample from the general population. On the basis of these findings, Lundh et al. (1994) concluded that perfectionism may serve as a predisposing factor for the development of persistent insomnia.

In a follow-up study, Vincent and Walker (2000) administered the FMPS, Hewitt and Flett’s Multidimensional Perfectionism Scale (HFMPS) (Hewitt et al., 1991), and a sleep questionnaire to a sample of 32 adults with chronic insomnia and a sample of 26 healthy controls. The HFMPS distinguishes between three dimensions of perfectionism: self-oriented perfectionism (tendency to set high standards for achievement and to criticize oneself for not meeting the standards), other-oriented perfectionism (tendency to have unrealistically high standards for other people), and socially prescribed perfectionism (tendency to perceive that other people hold unrealistically high standards for oneself). Using these two perfectionism scales, Vincent and Walker (2000) found that individuals with chronic insomnia obtained comparatively higher scores on the FMPS subscales concern over mistakes, doubts about action, parental criticism, and on the HFMPS subscale socially prescribed perfectionism. However, only parental criticism was positively associated with a sleep parameter, namely, self-rated sleep-onset latency. Similar results for HFMPS-defined perfectionism were obtained by Azevedo et al. (2009) in a sample of 1163 university students: They found that difficulties falling asleep and maintaining sleep were associated with higher scores on the subscale socially prescribed perfectionism.

More recently, three cross-sectional studies explored potential mediators in the relationship between perfectionism and sleep disturbance. Brand et al. (2015) administered the FMPS and two sleep questionnaires to 346 university students. In line with previous investigations, insomnia severity was associated with higher scores on the aggregated subscales “concern over mistakes and doubts about action,” “parental expectations and criticism,” and “personal standards.” A structural equation modeling analysis indicated that these associations were mediated by perceived stress, stress coping, emotion regulation, and mental toughness. In the second study, Akram et al. (2017) compared 39 individuals with DSM-5 defined insomnia and 39 normal sleepers and found that the insomnia group obtained higher scores on three FMPS subscales: concern over mistakes, doubts about action, and parental criticism. The associations between these facets of perfectionism and insomnia were partially mediated by symptoms of anxiety. Depressive symptoms were also correlated with these facets of perfectionism and insomnia but did not mediate between them. In the third study, Lin et al. (2017) administered the Almost Perfectionism Scale-Revised (APSR) (Slaney et al., 2001) and a sleep questionnaire to 1,664 adolescent students. The APSR distinguishes between three dimensions of perfectionism: high standards or adaptive perfectionism (tendency to set high standards for oneself), discrepancy or maladaptive perfectionism (tendency to perceive that personal high standards are not being met), and order (tendency to appreciate order, organization, neatness, and discipline). Using this distinction, Lin et al. (2017) found that insomnia severity was associated with higher scores on maladaptive perfectionism and that this association was mediated by worry and rumination.

In the only study to date to use objective measures of sleep, Johann et al. (2017) observed that the FMPS subscales concern over mistakes, personal standards, parental expectations, and parental criticism were associated with polysomnographic markers of poor sleep in the first sleep laboratory night, in particular with the number of nocturnal awakenings. These results corroborate and extend the questionnaire-based findings on the relations between perfectionism and sleep disturbance.

Further evidence for a link between perfectionism and insomnia was provided by four longitudinal studies. In the first study, Jansson-Fröjmark and Linton (2007) twice administered, with a temporal distance of 1 year, two scales of the FMPS (concern over mistakes and personal standards; Frost et al., 1990) and two sleep questionnaires to a sample of 1,936 participants from the general population. To assess emotional distress, the investigators also asked the participants to complete the Hospital Anxiety and Depression Scale (HADS) (Zigmond and Snaith, 1983). Regression analyses indicated that concern over mistakes was associated with existing and future insomnia. However, when emotional distress was controlled for, these associations became statistically non-significant. This pattern of results is consistent with the idea that the association between perfectionism and insomnia was mediated by emotional distress (anxiety and depression) although mediation was not directly tested by Jansson-Fröjmark and Linton (2007) (for a discussion of this finding, see Akram et al., 2015). In the second longitudinal study, Azevedo et al. (2010) administered the HFMPS (Hewitt et al., 1991) and two sleep items to a sample of 870 students. At follow-ups after 1 and 2 years, 592 and 305 students, respectively, completed the questionnaires once again. Regression analyses revealed that the dimension of socially prescribed perfectionism predicted difficulties falling asleep and maintaining sleep at the two follow-ups [see also Maia et al. (2011), who analyzed the same dataset].

In the third longitudinal study, Lombardo et al. (2013) administered the FMPS, the Achenbach Depression Scale (ASR) (Achenbach et al., 2005), and a sleep questionnaire to a community sample of 819 adults, 720 of whom completed the ASR and the sleep questionnaire again 3 months later. Analyses using structural equation modeling indicated that, both cross-sectionally and longitudinally, insomnia symptoms were associated with higher depression scores and that the statistical effect of insomnia on depression was partially mediated by concern over mistakes and doubts about action. In the fourth longitudinal study, Akram et al. (2015) administered the FMPS and the HFMPS to a sample of 76 members of the general population and found that baseline insomnia severity was associated with higher scores on the subscales doubts about action and parental criticism at follow-up after 12 months. Anxiety as assessed with the HADS (Zigmond and Snaith, 1983) partially mediated the associations between baseline insomnia severity and future perfectionism. Of note, both anxiety and depression partially mediated the association between perfectionism and insomnia in a cross-sectional analysis at baseline.

Taken together, five main findings may be gathered from the previously mentioned studies: (1) Insomnia symptoms are associated with the FMPS dimension concern over mistakes (Lundh et al., 1994; Johann et al., 2017) and the HFMPS dimension socially prescribed perfectionism (Vincent and Walker, 2000; Azevedo et al., 2009), which are highly correlated (Frost et al., 1993); (2) insomnia symptoms are also associated with the FMPS dimension doubts about action (Lundh et al., 1994; Akram et al., 2017); (3) the relations between insomnia symptoms and perfectionism are most likely bidirectional (Azevedo et al., 2010; Akram et al., 2015); (4) cognitive arousal (worry and rumination; Lin et al., 2017), affective arousal (anxiety: Akram et al., 2017; depression: Akram et al., 2015 [cross-sectional analysis]; perceived stress: Brand et al., 2015), and emotion regulation (Brand et al., 2015) seem to play a mediating role between perfectionism and insomnia symptoms; and (5) anxiety may also play a mediating role between insomnia symptoms and future perfectionism (Akram et al., 2015), while perfectionism, in turn, seems to play a mediating role between insomnia symptoms and depression (Lombardo et al., 2013).

Independently of insomnia, research has revealed that perfectionism, in particular the two previously mentioned dimensions concern over mistakes and socially prescribed perfectionism, is linked to a tendency to engage in counterfactual thinking and to experience counterfactual emotions (Tangney, 2002; Sirois et al., 2010; Flett et al., 2016; Stoeber and Diedenhofen, 2017). Counterfactual thinking refers to comparisons between the facts of reality (e.g., actual performance) and counterfactual imaginations of what might have been (e.g., ideal performance) (Epstude and Roese, 2008). Discrepancies between actual and ideal behavior typically elicit counterfactual emotions, such as regret, shame, and guilt, which are associated with the cognitive appraisal “you wish you had not done something, or that you could undo it” (Frijda et al., 1989). The available evidence suggests that perfectionists, especially those who are very concerned about their mistakes and the high standards imposed upon them by others, frequently review their own behavior, engage in counterfactual comparisons and self-exhortations (e.g., “I should [not] have done that!”), and correspondingly experience negative emotions, in particular shame and guilt (Frost et al., 1990, 1997; Tangney, 2002; Fedewa et al., 2005; Stoeber et al., 2008; Sirois et al., 2010).

Given that bedtime may often be the first quiet period in the course of the day available to review one’s own behavior, we recently proposed that this time window might be particularly suitable for the emergence of counterfactual thinking and associated feelings of regret, shame, and guilt (Schmidt and Van der Linden, 2009). In support of this hypothesis, we found that university students reported often experiencing counterfactual thoughts and emotions at bedtime and that the frequency of such thoughts and emotions was linked to insomnia severity (Schmidt and Van der Linden, 2009). Similar findings were obtained in a sample of elderly people, whose age ranged from 51 to 98 years (Schmidt et al., 2011b). The pattern of their responses clearly suggested daytime variation in the experience of counterfactual emotions: While regret frequency remained at comparatively low levels for most of the waking hours, a sharp rise occurred in the evening after going to bed. Additional evidence for a link between counterfactual emotions and insomnia has been found in samples of healthcare providers (Courvoisier et al., 2011; Schmidt et al., 2015) and of adolescents with bipolar disorder (Roybal et al., 2011).

Of note, we previously found that experimental activation of regret delays sleep onset in individuals with habitually high levels of regret independently of preexisting levels of anxiety and insomnia (Schmidt and Van der Linden, 2013). This finding suggests that counterfactual emotions may contribute to sleep disturbance over and above the well-established effect of anxiety (Mitchell et al., 2012).

In the present study, we therefore focused on counterfactual emotions as a specific pathway whereby perfectionism may influence sleep. Specifically, we hypothesized that perfectionistic individuals, in particular those scoring high on concern over mistakes, would be prone to engage in counterfactual processing at bedtime and to experience the corresponding emotions of regret, shame, and guilt. As a consequence, their sleep should be impaired. To test this hypothesis, we administered questionnaires on perfectionism, counterfactual processing at bedtime, and insomnia severity to a sample of university students.

## Materials and Methods

### Participants and Procedure

One hundred eighty undergraduate students of psychology participated in this study to fulfill a course requirement. The sample comprised 153 women and 27 men, aged 18–44 (*M* = 21.85; *SD* = 3.56). On the occasion of a larger data collection session for undergraduate students in psychology, participants were asked to complete the three questionnaires that are described in detail below. They were assessed in groups of up to 65 people, with questionnaires being administered in paper-and-pencil versions. At the beginning of the data collection session, the students were informed that data collection was fully anonymous, that participation was voluntary, and that the course requirement could be fulfilled with other activities. The participants then provided informed written consent according to the ethical guidelines of the Swiss Society of Psychology. The procedure for the data collection session was approved by the Ethics Commission of the Faculty of Psychology and Educational Sciences of the University of Geneva.

### Questionnaires

#### Frost Multidimensional Perfectionism Scale (FMPS)

To assess individual differences in perfectionism, we administered the French version (Rhéaume et al., 1995) of the FMPS (Frost et al., 1990). The FMPS is composed of 35 items that are rated on a 5-point Likert scale ranging from 1 (*strongly disagree*) to 5 (*strongly agree*). On the basis of the item scores, six subscale scores reflecting distinct domains of perfectionism can be computed: concern over mistakes (e.g., “People will probably think less of me if I make a mistake”), doubts about actions (e.g., “Even when I do things very carefully, I often feel that it is not quite right”), personal standards (e.g., “Other people seem to accept lower standards than I do”), parental expectations (e.g., “Only outstanding performance is good enough in my family”), parental criticism (e.g., “I never felt like I could meet my parents’ standards”), and organization (e.g., “I try to be an organized person”). Internal consistency of the subscales of the French FMPS has been satisfactory across different populations, with Cronbach’s alpha ranging from 0.60–0.90 (Bouvard, 2009).

#### Bedtime Counterfactual Processing Questionnaire (BCPQ)

To gauge individual differences in the frequency of counterfactual thoughts and emotions during the pre-sleep period, we used the BCPQ (Schmidt and Van der Linden, 2009). The questions are preceded by the following instruction: “When in bed in the evening, one sometimes reviews the day that has just come to an end. Hereafter, please indicate how often different kinds of thoughts occur to you as you are trying to get to sleep.” In the present study, we used an extended version of the original BCPQ that included three new items (numbered 3, 6, and 9 hereafter) designed to control for response bias effects (Schmidt and Van der Linden, 2011). These items assess pride-related experiences, quite the opposite of feelings of regret, shame, and guilt.

The wording of the 10 items is as follows (Schmidt and Van der Linden, 2011, pp. 21–22): “(1) “After going to bed, how often do you regret your behavior toward others?”; (2) “After going to bed, how often do you think: ‘If only I had made another choice!”’; (3) “After going to bed, how often do you feel proud of yourself when you look back at all that you accomplished?”; (4) “After going to bed, how often do you feel guilty because you have the impression of having done wrong to others?”; (5) “After going to bed, how often do you think: ‘If only I were more prudent!”’; (6) “After going to bed, how often do you congratulate yourself for the way you treated other people?”; (7) “After going to bed, how often do you feel ashamed of yourself because of your behavior?”; (8) “After going to bed, how often do you imagine how you would have liked to behave, but did not?”; (9) “After going to bed, how often do you feel happy with the way you handled a conflict with other people?”; and (10) “After going to bed, how often do you feel preoccupied with the consequences of your behavior toward others?” Answers are given on a 5-point Likert scale ranging from 0 (*almost never*) to 4 (*very often*).” The original BCPQ (without the three control items) and the extended version have been shown to capture a single dimension and to possess high internal consistency, with Cronbach’s alpha ranging from 0.81 to 0.87.

#### Insomnia Severity Index (ISI)

To measure levels of sleep impairment during the month preceding the experiment, we used the French version of the ISI (Blais et al., 1997; Morin et al., 2011). The ISI comprises seven items that are rated on a 5-point Likert scale ranging from 0 (*not at all*) to 4 (*extremely*). Respondents are asked to evaluate the following dimensions of insomnia: (a) severity of insomnia during the past month (difficulty falling asleep, difficulty staying asleep, problem waking up too early); (b) satisfaction with current sleep patterns; (c) interference with daytime functioning; (d) noticeability of impairment to significant others; and (e) level of distress caused by the sleep problem. Total scores range from 0 to 28, with higher scores indicating higher perceived insomnia severity. The French version of the ISI has been shown to possess sound internal consistency (α = 0.88) and to correlate strongly (*r* = 0.67) with the PSQI (Buysse et al., 1989; Blais et al., 1997). Concurrent validity of the ISI has also been demonstrated using other-administered versions of this instrument, polysomnography, and cardiovascular measures (Bastien et al., 2001; Schmidt et al., 2010b). To complement the ISI, two questions of the PSQI were used in the present study: Item 5h (“During the past month, how often have you had trouble sleeping because you had bad dreams?”) and Item 7 (“During the past month, how often have you taken medicine [prescribed or ‘over the counter’] to help you sleep?”). Answers to these items are given on a 4-point Likert scale ranging from 0 (*not during the past month*) to 3 (*three or more times a week*).

## Results

### Preliminary Analyses

For the three questionnaires described in detail earlier, Cronbach’s alpha coefficients, mean scores, and standard deviations are provided in **Table 1**. The range of alpha coefficients (0.74–0.89) indicates that the three questionnaires possess sound internal consistency. According to the norms for the ISI provided by Bastien et al. (2001), 30.7% of our participants did not show any sign of clinically significant insomnia prior to the experiment (score range [Bastien et al., 2001] = 0–7), 48.3% gave evidence of subthreshold insomnia (range = 8–14), and 14.4% could be considered as suffering from moderate clinical insomnia (range = 15–21), whereas no participant obtained scores corresponding to severe clinical insomnia (range = 22–28). Regarding the two PSQI questions, 44.4% of our participants reported not having had trouble sleeping as a result of bad dreams during the past month, whereas 38.3% indicated having had such problems less than once a week, 13.3% once or twice a week, and 3.9% three or more times a week. Finally, 4.4% of our participants reported having taken medicine to help them sleep less than once a week during the past month, 2.2% once or twice a week, and 2.2% three or more times a week.

**Table 1:** Cronbach’s alpha coefficients, means, and standard deviations for the questionnaire scores.

Variables	α	*M*	*SD*
FMPS Concern over mistakes	0.88	20.38	7.19
FMPS Doubts about actions	0.74	10.27	3.39
FMPS Personal standards	0.85	20.32	5.67
FMPS Parental expectations	0.86	11.31	4.48
FMPS Parental criticism	0.81	7.06	3.21
FMPS Organization	0.89	22.83	4.59
BCPQ Total score	0.82	9.44	4.65
ISI Total score	0.82	8.34	5.12

*FMPS, Frost Multidimensional Perfectionism Scale; BCPQ, Bedtime Counterfactual Processing Questionnaire; ISI, Insomnia Severity Index.*

### Correlation Analyses

To explore the associations between the questionnaire scores, we computed Pearson correlations, which are shown in **Table 2**. Results revealed that four dimensions of perfectionism, as assessed by the FMPS, were significantly associated with counterfactual thoughts and emotions at bedtime, as measured by the BCPQ: Concern over mistakes (*r* = 0.31, *p* < 0.001) and doubts about action (*r* = 0.42, *p* < 0.001) were positively related to counterfactual processing, whereas organization (*r* = −0.28, *p* < 0.001) and parental expectations (*r* = −0.15, *p* < 0.05) showed negative correlations. A similar pattern of correlations emerged for insomnia severity, as evaluated by the ISI: Concern over mistakes (*r* = 0.23, *p* < 0.01) and doubts about action (*r* = 0.27, *p* < 0.001) were positively related to insomnia severity, whereas organization (*r* = −0.19, *p* < 0.05) was negatively correlated to it. Finally, in accordance with previous research (Schmidt and Van der Linden, 2009; Schmidt et al., 2011b), the frequency of counterfactual thoughts and emotions at bedtime was positively related to insomnia severity (*r* = 0.31, *p* < 0.001).

**Table 2:** Pearson correlations between questionnaire scores.

		1	2	3	4	5	6	7	8
1	FMPS Concern over mistakes	1							
2	FMPS Doubts about action	0.47^∗∗∗^	1						
3	FMPS Personal standards	0.53^∗∗∗^	0.14	1					
4	FMPS Parental expectations	0.23^∗∗^	0.01	0.34^∗∗∗^	1				
5	FMPS Parental criticism	0.27^∗∗∗^	0.27^∗∗∗^	0.10	0.67^∗∗∗^	1			
6	FMPS Organization	−0.04	−0.18^∗^	0.22^∗∗^	0.08	−0.09	1		
7	BCPQ	0.31^∗∗∗^	0.42^∗∗∗^	0.04	−0.15^∗^	0.06	−0.28^∗∗∗^	1	
8	ISI	0.23^∗∗^	0.27^∗∗∗^	0.04	0.03	0.11	−0.19^∗^	0.35^∗∗∗^	1

*FMPS, Frost Multidimensional Perfectionism Scale; BCPQ, Bedtime Counterfactual Processing Questionnaire; ISI, Insomnia Severity Index. ^∗∗∗^Significant at the 0.001 level, two-tailed; ^∗∗^significant at the 0.01 level, two-tailed; ^∗^significant at the 0.05 level, two-tailed.*

Of note, the frequency of sleep-interfering dreams, as measured by item 5 h of the PSQI, was also positively related to concern over mistakes (*r* = 0.18, *p* < 0.05), doubts about action (*r* = 0.17, *p* < 0.05), counterfactual processing at bedtime (*r* = 0.25, *p* < 0.001), and insomnia severity (*r* = 0.40, *p* < 0.001). In line with earlier research (Schmidt and Van der Linden, 2009) suggesting that counterfactual processing may continue during sleep and manifest itself in dream mentation, these correlations indicate that perfectionism may contribute to such processes.

### Mediation Analysis

**FIGURE 1:**
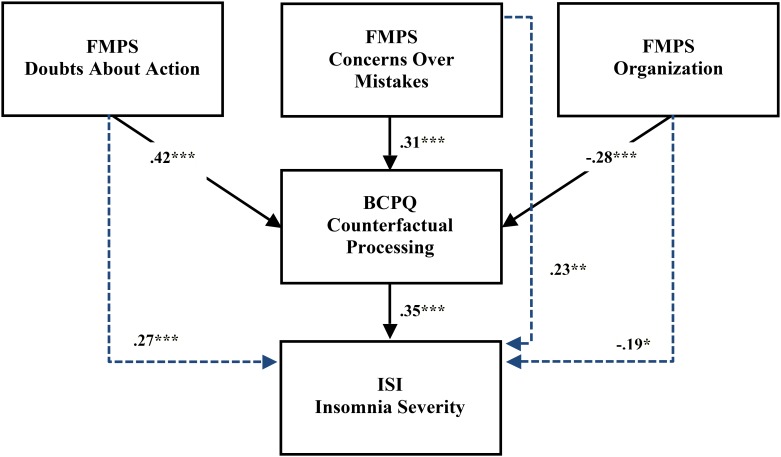
Schematic illustration of the relations between three dimensions of perfectionism (concern over mistakes, doubts about action, and organization), the frequency of counterfactual thoughts and emotions at bedtime, and insomnia severity. Indices represent standardized coefficients for simple regressions between the variables (^∗^*p* < 0.05; ^∗∗^*p* < 0.01; ^∗∗∗^*p* < 0.001). Dashed lines represent simple regression coefficients that became statistically non-significant in the mediation analysis. FMPS, Frost Multidimensional Perfectionism Scale; BCPQ, Bedtime Counterfactual Processing Questionnaire; ISI, Insomnia Severity Index.

To explore whether the frequency of counterfactual thoughts and emotions at bedtime mediated the effects of perfectionism on insomnia severity, we computed a series of regression analyses (MacKinnon et al., 2007). First, in light of the results of the correlation analyses, insomnia severity was separately regressed on the following three dimensions of perfectionism: concern over mistakes, doubts about action, and organization. Mirroring the correlation analyses, concern over mistakes (*B* = 0.16, *t* = 3.12, *p* < 0.01, adj. *R*^2^ = 0.05), doubts about action (*B* = 0.40, *t* = 3.68, *p* < 0.001, adj. *R*^2^ = 0.07) and organization (*B* = −0.20, *t* = −2.41, *p* < 0.05, adj. *R*^2^ = 0.03) all predicted insomnia severity. Second, the effect of counterfactual processing on insomnia severity was assessed, which was also significant (*B* = 0.39, *t* = 5.04, *p* < 0.001, adj. *R*^2^ = 0.12). In a third step, insomnia severity was again regressed on each of the three dimensions of perfectionism separately, this time controlling for counterfactual processing as mediator. As might be expected in the case of complete mediation, regression coefficients became non-significant for concern over mistakes (*B* = 0.09, *t* = 1.76, *p* = 0.08, total adj. *R*^2^ = 0.13), doubts about action (*B* = 0.22, *t* = 1.88, *p* = 0.06, total adj. *R*^2^ = 0.13), and organization (*B* = −0.10, *t* = −1.20, *p* = 0.23, total adj. *R*^2^ = 0.12) (see **Figure 1**).

To test whether these mediation effects were statistically significant, we used the Sobel coefficient, computed as the difference (c-c′) between (1) the regression coefficient for insomnia severity on the independent variable (c), and (2) the same regression coefficient, this time adjusted for the mediator (c′). Confidence intervals (CIs) for each Sobel test coefficient were estimated using bootstrap with 10,000 samples. Results for the three predictors were as follows: concern over mistakes: c-c′ = 0.07, 95% CI: [0.03; 0.13]; doubts about action: c-c′ = 0.18, 95% CI: [0.09; 0.32]; and organization: c-c′ = −0.10, 95% CI: [−0.18; −0.05]. These results indicate that the frequency of counterfactual thoughts and emotions at bedtime significantly mediated the effect of the three dimensions of perfectionism on insomnia severity.

## Discussion

Previous investigations have suggested that specific facets of perfectionism are associated with sleep disturbance, both in the general population and in individuals with insomnia (see **Table 3**). However, the precise processes whereby perfectionism may influence sleep have as yet remained largely unexplored. The present study was designed to elucidate these processes by examining the role of counterfactual processing. The main findings suggest that two dimensions of perfectionism, namely, concern over mistakes and doubts about action, are positively correlated with insomnia severity and that the frequency with which counterfactual thoughts and emotions (regret, shame, and guilt) are experienced at bedtime mediates the effect of perfectionism on sleep.

**Table 3:** Overview: empirical studies of perfectionism and insomnia.

Author (Year)	Sample	Measures	Main findings
Akram et al., 2015	Members of the general population (*N* = 76 at baseline; *N* = 57 after 12 months)	FMPS^a^ HFMPS^b^ ISI^c^	Baseline insomnia severity was associated with higher scores on the subscales doubts about action and parental criticism at follow-up (12 months later). This relationship was partially mediated by baseline anxiety.
Akram et al., 2017	Individuals with insomnia disorder (*N* = 39) and normal sleepers (*N* = 39)	FMPS HFMPS	Compared to normal sleepers, individuals with insomnia displayed higher scores on the subscales concern over mistakes, doubts about action, and parental criticism. These differences were partially mediated by anxiety.
Azevedo et al., 2009	University students (*N* = 1163)	HFMPS 2 sleep items	Difficulties falling asleep and maintaining sleep were associated with higher scores on the subscale socially prescribed perfectionism. In female participants, difficulties falling asleep and maintaining sleep were also associated with higher scores on the subscale self-oriented perfectionism.
Azevedo et al. (2010)	University students (*N* = 870 at baseline; *N* = 592 after 1 year; *N* = 305 after 2 years)	HFMPS 2 sleep items	Difficulties falling asleep and maintaining sleep at 2 follow-ups (after 1 and 2 years) were associated with higher scores on the subscale socially prescribed perfectionism.
Brand et al., 2015	University students (*N* = 346)	FMPS ISI PSQI^d^	Insomnia severity was associated with higher scores on the aggregated subscales concern over mistakes and doubts about action, parental expectations and criticism, and personal standards. These associations were mediated by perceived stress, stress coping, emotion regulation, and mental toughness.
Jansson-Fröjmark and Linton, 2007	Random sample of the general population (*N* = 1936)	FMPS (2 subscales) BNSQ^e^ USI^f^	Insomnia severity at baseline and at follow-up (12 months later) was related to higher baseline scores on the subscale concern over mistakes. These associations were no longer significant when baseline emotional distress (anxiety and depression) were controlled for.
Johann et al., 2017	Unselected sleep laboratory patients (*N* = 334)	FMPS PSG^g^	The FMPS total score was associated with the number of nocturnal awakenings on the first sleep laboratory night. The subscales concern over mistakes, personal standards, parental expectations, and parental criticism were significantly associated with markers of poor sleep.
Lin et al., 2017	Adolescent students (*N* = 1664)	APSR^h^ PSQI	Insomnia severity was associated with higher scores on the subscale discrepancy (maladaptive perfectionism). The association between maladaptive perfectionism and insomnia was mediated by worry and rumination.
Lombardo et al., 2013	Community sample of adults (*N* = 819 at baseline; *N* = 720 after 3 months)	FMPS PSQI	Insomnia severity was associated with higher depression scores at baseline and at follow-up (after 3 months). This association was partly mediated by concern over mistakes and doubts about action (both measured only at baseline).
Lundh et al., 1994	Study 1: random sample of the general population, age- and gender-stratified (*N* = 383) Study 2: patients with persistent insomnia (*N* = 70)	FMPS BNSQ USI	Study 1: Insomnia severity was associated with a higher FMPS total score and with higher scores on the subscales concern over mistakes, doubts about action, and personal standards. Study 2: Compared to normal sleepers, insomnia patients obtained higher scores on the subscales concern over mistakes, doubts about action, and personal standards.
Vincent and Walker, 2000	Adults with chronic insomnia (*N* = 32) and healthy controls (*N* = 26)	FMPS HFMPS PSQI	Compared to healthy controls, adults with chronic insomnia obtained higher scores on the FMPS subscales concern over mistakes, doubts about action, and parental criticism. Among participants with chronic insomnia, parental criticism was associated with the PSQI subscale sleep-onset latency.

*^a^Frost Multidimensional Perfectionism Scale. ^b^Hewitt and Flett’s Multidimensional Perfectionism Scale. ^c^Insomnia Severity Index. ^d^Pittsburgh Sleep Quality Index. ^e^Basic Nordic Sleep Questionnaire. ^f^Uppsala Sleep Inventory. ^g^Polysomnography. ^h^Almost Perfectionism Scale-Revised.*

As outlined in the introduction, current etiological models of insomnia assign a central role to hyperarousal, which may be further differentiated into its cognitive, emotional, and physiological components (Espie, 2002, 2007; Harvey, 2002; Riemann et al., 2010). Within this theoretical framework, personality traits, such as the dimensions of perfectionism, may be understood as factors that predispose people to experience arousal more easily, more intensely, or for a longer time in response to stressful life events (Spielman and Glovinsky, 1991; Heller, 1993). The findings of this study indicate that the two previously mentioned dimensions of perfectionism may function as predisposing factors in the sense described. Specifically, people scoring high on concern over mistakes and doubts about action are prone to frequently compare their actual behavior with imagined ideal behavior. As a consequence of perceived shortcomings, these individuals are liable to experience negatively valenced counterfactual emotions, such as regret, shame, and guilt, especially at bedtime, which has previously been shown to be a preferential time window for counterfactual processing (Schmidt et al., 2011b). The resulting state of emotional arousal then interferes with sleep, as the data of the current study and previous investigations (Schmidt and Van der Linden, 2009; Schmidt et al., 2011b; Čapková et al., 2018) concurrently suggest. Taken together, these studies add to the growing literature highlighting the importance of affective processes in insomnia (Baglioni et al., 2010; Schmidt et al., 2011a).

An unexpected finding was that the dimension of perfectionism termed organization was negatively related both to the frequency of counterfactual thoughts and emotions at bedtime and to insomnia severity. Put differently, this dimension of perfectionism may thus act as a protective factor against the experience of sleep-interfering counterfactual processing. This result accords with previous research suggesting that organization, along with personal standards, belongs to a cluster of adaptive aspects of perfectionism that are positively linked to achievement striving and efficacy, whereas they are negatively linked to procrastination and aversion to completing important tasks (Frost et al., 1993). In contrast, concern over mistakes and doubts about action belong to a cluster of maladaptive aspects of perfectionism that are linked to procrastination, obsessive-compulsive symptoms, self-critical depression, worry, and rumination (Lin et al., 2017). In light of these previous lines of research, our finding may tentatively be interpreted in the sense that well-organized individuals are more likely to complete important tasks and finish related thought processes before retiring for the night, rendering them less prone to experience sleep-interfering counterfactual emotions. This interpretation chimes with a behavioral treatment strategy for insomnia that involves scheduling a time-limited period to worry every day, with the aim to minimize worrying at bedtime (Fiorentino et al., 2010).

Although our study provides new insights into the processes whereby perfectionism may interfere with sleep, several limitations have to be considered when drawing conclusions from our data. First, the design of our study is cross-sectional, which precludes causal inferences. However, the case for a negative effect of perfectionism on sleep is strengthened by the earlier presented findings from two longitudinal investigations (Jansson-Fröjmark and Linton, 2007; Azevedo et al., 2010) (see **Table 3**). Second, our study was conducted with a sample of university students. Given that the opportunities for reparative action generally decline with age, the intensity of counterfactual emotions is liable to increase across the lifespan and to have progressively stronger effects on sleep (Wrosch et al., 2005; Schmidt and Van der Linden, 2011). Therefore, a fruitful avenue for future research might be to compare the relations between perfectionism, counterfactual emotions, and sleep in younger and older adults. It will also be important to study these processes in clinical samples of individuals with diagnosed insomnia in order to determine the roles that perfectionism and counterfactual processing play in the transition from acute to chronic forms of sleep disturbance – for this transition, see Ellis et al. (2012). Third, our study focused on the impact of negative emotions deriving from past experiences. However, it is well established that future events (e.g., task of giving a speech, Gross and Borkovec, 1982) and positive emotions (e.g., romantic love, Brand et al., 2007) can also interfere with sleep. Regarding perfectionism, it seems particularly promising to explore a potential interaction between stressful future events and the dimension of doubts about action. Fourth, our sample comprised 153 women and only 27 men, thereby precluding statistical explorations of potential gender differences in perfectionism and insomnia. Fifth, our total sample was comprised of 180 participants. The fact that we obtained statistically significant results suggests that the sample size yielded sufficient power. However, a replication of our findings in a larger sample, ideally using a longitudinal design, is clearly warranted to buttress our conclusions and to gain a more fine-grained understanding of the reciprocal relations between perfectionism and sleep.

A final limitation of our study results from the use of questionnaires. Future research on perfectionism and sleep may complement self-report measures with behavioral and physiological measures. An illustration of such an approach is provided by Spiegelhalder et al. (2012), who used punctuality as a behavioral indicator of perfectionism and found strong relationships between this indicator and polysomnographic sleep parameters (e.g., sleep duration, wake after sleep onset, sleep efficiency). Moreover, observational studies may be complemented with experimental designs. As mentioned earlier, Schmidt and Van der Linden (2013) found that experimental activation of regret delays sleep onset when compared to activation of pride or of neutral working day schedules. A promising perspective for future studies would be to experimentally compare the effects of counterfactual emotions on sleep in individuals scoring high or low on different dimensions of perfectionism.

Given that different personality traits of the internalizing and externalizing spectrums may predispose for sleep disturbance, an important challenge for future research will be to develop integrative conceptualizations of how these traits can interact in the etiology of insomnia. For instance, several previous studies suggest that a specific facet of impulsivity, urgency, adversely affects sleep through an increase of counterfactual thoughts and emotions at bedtime (Schmidt and Van der Linden, 2009; Schmidt et al., 2011b). Impulsive urgency can be defined as the tendency to act rashly, especially under conditions of negative affect (Whiteside and Lynam, 2001). To account for the sleep-related findings, Schmidt and Van der Linden (2011) argued that people scoring high on impulsive urgency are prone to frequently run into situations or adopt behaviors that evoke counterfactual emotions, such as regret, shame, and guilt. As a consequence, they are liable to experience more counterfactual emotions at the end of the day as they try to get to sleep. When combined with the findings from the present study, one might speculate that high urgency individuals with perfectionistic tendencies should be most susceptible to the experience of sleep-interfering counterfactual processing. At first sight, the combination of overcontrolled personality traits, such as perfectionism, and undercontrolled traits, such as impulsivity, seems paradoxical. However, recent research revealed that this combination may play a powerful pathogenic role, for instance in eating disorders (Boone et al., 2014). In view of this line of research, the intriguing hypothesis of a combined impact of impulsivity and perfectionism on sleep awaits further investigation.

As to treatment implications, inspirations for the development of sleep-promoting interventions for perfectionistic individuals may be drawn from three sources: the literatures on perfectionism, self-compassion and self-forgiveness, and counterfactual thinking. Concerning the literature on perfectionism, cognitive-behavioral intervention techniques have been devised that use diverse tools (e.g., thought records, surveys, and behavioral experiments) to modify factors that maintain maladaptive perfectionism, such as cognitive biases (e.g., selective focus on failures) and counterproductive behaviors – e.g., procrastination; for a review, see Egan et al. (2011). For the second strand of literature, it has been shown that fostering self-compassion and self-forgiveness may counteract adverse effects of excessive self-criticism that is typical of maladaptive perfectionism (Frost et al., 1997; Ingersoll-Dayton and Krause, 2005; Gilbert and Procter, 2006). Regarding the third strand of literature, research suggests that individuals scoring high on maladaptive dimensions of perfectionism tend to engage in counterproductive forms of counterfactual thinking in reaction to negative events (Sirois et al., 2010). For instance, they are comparatively more likely to develop subtractive (antecedent elements removed to alter reality) rather than additive counterfactuals (antecedent elements added to alter reality). Crucially, additive counterfactuals have the potential to highlight novel options that increase the chances of creative problem solving (Markman et al., 2007). Considering the increasing prevalence of perfection among young populations (Curran and Hill, 2017), more research is clearly needed to explore whether promoting productive forms of counterfactual thinking, possibly in combination with the previously mentioned intervention techniques, is apt to diminish sleep-interfering counterfactual processing in perfectionistic individuals.

## Author Contributions

RS and MVdL designed the study. RS and DC conducted the statistical analyses. All authors participated in the interpretation of the data and in drafting the manuscript.

## Conflict of Interest Statement

The authors declare that the research was conducted in the absence of any commercial or financial relationships that could be construed as a potential conflict of interest.
